# Myelination Is Associated with Processing Speed in Early Childhood: Preliminary Insights

**DOI:** 10.1371/journal.pone.0139897

**Published:** 2015-10-06

**Authors:** Nicolas Chevalier, Salome Kurth, Margaret Rae Doucette, Melody Wiseheart, Sean C. L. Deoni, Douglas C. Dean, Jonathan O’Muircheartaigh, Katharine A. Blackwell, Yuko Munakata, Monique K. LeBourgeois

**Affiliations:** 1 Department of Psychology, University of Edinburgh, Edinburgh, United Kingdom; 2 Department of Integrative Physiology, University of Colorado Boulder, Boulder, Colorado, United States of America; 3 Department of Psychology, York University, Toronto, Ontario, Canada; 4 Brown University School of Engineering, Providence, Rhodes Island, United States of America; 5 Department of Neuroimaging, King’s College London, Institute of Psychiatry, London, United Kingdom; 6 Department of Psychology, Salem College, Winston-Salem, North Carolina, United States of America; 7 Department of Psychology and Neuroscience, University of Colorado Boulder, Boulder, Colorado, United States of America; University of Pennsylvania, UNITED STATES

## Abstract

Processing speed is an important contributor to working memory performance and fluid intelligence in young children. Myelinated white matter plays a central role in brain messaging, and likely mediates processing speed, but little is known about the relationship between myelination and processing speed in young children. In the present study, processing speed was measured through inspection times, and myelin volume fraction (VF_M_) was quantified using a multicomponent magnetic resonance imaging (MRI) approach in 2- to 5-years of age. Both inspection times and VF_M_ were found to increase with age. Greater VF_M_ in the right and left occipital lobes, the body of the corpus callosum, and the right cerebellum was significantly associated with shorter inspection times, after controlling for age. A hierarchical regression showed that VF_M_ in the left occipital lobe predicted inspection times over and beyond the effects of age and the VF_M_ in the other brain regions. These findings are consistent with the hypothesis that myelin supports processing speed in early childhood.

## Introduction

Infants’ cognitive capacities increase in complexity with age, ensuring increasing adaptiveness to the environment throughout childhood. An important aspect of cognitive development is the speed with which children can perform mental operations, a critical characteristic of the information-processing system [1]. Gains in processing speed increase the amount of information that can be mentally manipulated and the complexity of such manipulations [2–4]. Therefore, gains in processing speed have a substantive impact on cognitive changes through developmental cascades during childhood; that is, processing speed increases lead to improvements in working memory, which in turn yield benefits in intelligence [1–3,5,6]. Consistent with such cascades, children’s processing speed mediates the effect of age on working memory, inhibition, and arithmetical skills [5–9].

Childhood cognitive development is intertwined with brain structural maturation. White matter maturation steadily increases throughout childhood [6,10–12]. As the formation of the myelin sheath surrounding neuronal axons fasten neural impulse propagation, it is likely a major contributor to processing speed [13]. Indeed increasing processing speed is associated with white matter integrity during middle childhood and adolescence [14–17]. Similarly, white matter microstructure relates to children’s cognitive abilities further down the developmental cascades, such as inhibition [18], spatial working memory [19], arithmetic skills [20], and reading [21]. Beyond correlational evidence, reduced radial diffusivity within white matter pathways connecting the frontal lobes, and reduced mean diffusivity in frontal and occipital lobe white matter, have been found following cognitive training in adults [22,23].

White matter microstructure seems to support these cognitive abilities via processing speed. In a large sample of 8- to 68-year-old participants, age influenced white matter tract microstructure in the inferior frontal-occipital fasciculus, which in turn influenced processing speed, and processing speed impacted executive function, attention, spatial working memory and verbal ability [24]. Similarly, processing speed mediated the relationship between white matter microstructure and reasoning in another sample of 6- to 18-year-olds; no specific brain region was responsible for this cascade, suggesting that processing speed may be an emergent property of the whole brain white matter ([25], see also [26]).

Although previous studies speak to the role of myelin in processing speed during middle childhood and adolescence, they leave open the question of whether myelin similarly supports processing speed earlier in development. The paucity of findings in early childhood likely relates to the difficulty inherent to scanning young children. Yet, substantial cognitive changes occur at that age, in terms of both processing speed and other cognitive abilities such as working memory and executive function [5,27,28]. Executive function and processing speed are more closely intertwined in younger than older children, probably because children draw upon the same processes across a wider variety of activities [7,9]. This age progression suggests that processing speed plays an especially critical role in cognitive functioning in early childhood. Clarifying the neural underpinnings of processing speed is key to understand cognitive development in early childhood.

In the present article, we report preliminary findings supporting an association between myelin and processing speed in 2- to 5-year-old children. Unlike most prior studies, we did not use diffusion tensor imaging (DTI), a technique that yields measures influenced not only by changes in myelin content or structure, but also changes in the local architectural milieu, i.e., fiber architecture, density and coherence [29,30]. To provide a more specific measure of myelin content, we used a rapid multi-component relaxometry technique [31], termed mcDESPOT (multi-component Driven Equilibrium Single-pulse Observation of T1 and T2) [32]. Briefly, mcDESPOT decomposes the observed MRI signal into contributions from 3 discrete signal sources: the extra-cellular water, a non-exchanging water pool representative of the cerebral spinal fluid, and the water trapped between the lipid bilayers of the myelin sheath (myelin water). By fitting a 3-pool tissue model to appropriately acquired data, mcDESPOT provides a quantitative estimate of the relative volume fraction of the myelin water pool (termed the myelin volume fraction, VF_M_), which is a surrogate measure of myelin content. This method has been previously used to investigate white matter myelin developing in infants and young children, showing region-specific VF_M_ development trajectories in early childhood [32–35].

Precise evaluation of processing speed is just as critical. We elected to measure processing speed using inspection times [36], that is, the minimal visual presentation time needed to identify targets, rather than reaction times. Unlike other common measures of processing speed in early childhood, inspection times do not rely critically on response execution time, which tremendously vary in young children, and minimally tap executive demands (e.g., goal maintenance, information manipulation in working memory, motor response selection), hence reflecting processing speed more specifically [9]. We expected faster inspection times with age to be related to greater VF_M_, especially in posterior regions given that inspection times minimally reflect executive and motor abilities.

## Materials and Methods

### Participants

Study participants were 12 children between 37 and 68 months of age (*M* = 49 months, *SD* = 12 months). They had no personal or family history of mental disorders or other chronic medical conditions. In addition, they had no diagnosed developmental disability and no physical handicaps. To be included, they also had to be born within 38 and 42 weeks of gestation, with a birth weight of at least 5.5 lbs. In addition, participants’ standard scores on the Mullen Scales of Early Learning [37], a measure of motor and cognitive development, fell within the normal range (*M* = 101, *SD* = 15, range: 83–122), further suggesting that participants were typically developing children. Participating families were recruited through flyers, website advertising, and personal contact at community events. During a home visit, written informed consent was obtained from parents. The study procedures were approved by the University of Colorado Boulder IRB and performed according to the Declaration of Helsinki.

### Procedure

The study included two visits on separate days. During the first visit, an MRI scan was obtained in the evening during non-sedated sleep (n = 3) or while the child watched a movie (n = 9). To minimize the likelihood of the child moving, scan time and noise were reduced through the selection of age-specific acquisition parameters [33]. Additional passive measures were used to reduce noise, including a sound-insulating bore insert (Quiet Barrier HD Composite, UltraBarrier USA), MiniMuff noise attenuators (Natus, USA), and electrodynamic headphones (MR Confon, Germany). The second visit included a 30-minute cognitive assessment, which was completed in the morning and administered by the same researcher for all children. Parents were present for testing but were instructed not to interact with the child. The two visits were counterbalanced in order, and separated by no more than 2 weeks.

#### Inspection time

Processing speed was assessed using inspection times with an adapted standard procedure [36], utilizing a Tobii x50 eye tracker (Tobii Technologies, Sweden). Calibration was performed using an automatic 5-point procedure prior to assessment for each child. The software package E-Prime version 1.2 (Psychology Software Tools, Pittsburgh, USA) was used for stimulus presentation and response sampling. Inspection times correlate with other measures of processing speed in both children and adults [6], suggesting it is a good index of processing speed.

**Fig 1:**
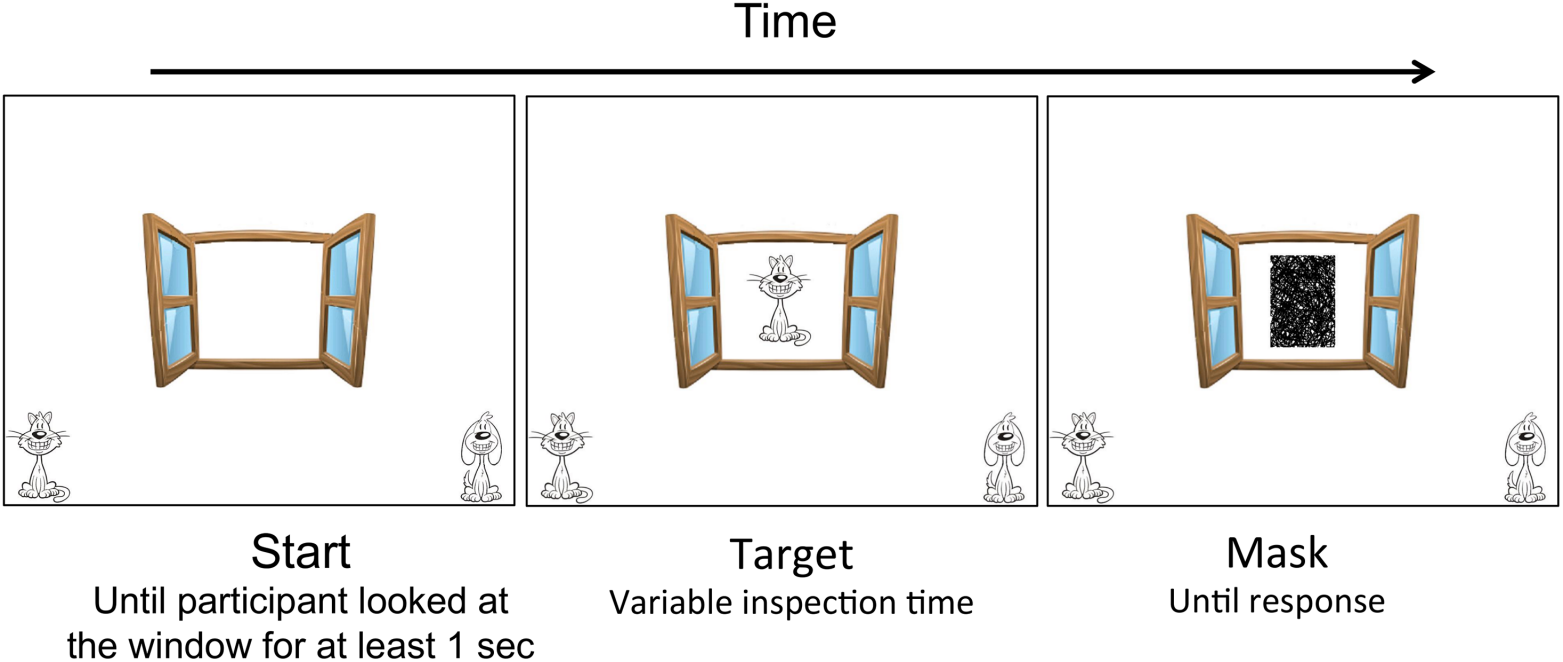
Computer screen presentations during the inspection time task. During a single trial, children were first presented with the target stimulus, followed by the black and white mask.

Prior to test trials, 6 practice trials were administered to ensure the child understood the task. The task included 30 trials with either an image of a cat or a dog, followed by a black and white mask (Fig 1). For the first test trial, target presentation time was 200 ms and followed an adapted staircase algorithm. This algorithm decreased presentation time by 17 ms after 2 correct responses and increased presentation time by 17 ms after a false response. The target and mask were presented at viewing distance of 60 cm. During presentation of the mask, children verbally reported which target had appeared or pointed to the corresponding picture at the bottom of the computer screen. Responses were recorded by the experimenter. The succeeding trial was initiated following the child’s response and after the eye tracker captured the child’s gaze for at least 1000 ms. This ensured that the child was looking at the computer screen during presentation of the target. Inspection times were calculated as the mean presentation time over the trials following the shortest presentation time for which children correctly identified the target on 2 successive trials. Log-transformed inspection times were used as dependent variable. On average, 12 trials were used in the calculation (*SD* = 10). As expected, there was a negative correlation between the number of trials in the calculation and age, *r* = -.716, *p* = .009. Older children needed less time to process the target, which required more trials for presentation time to progressively decrease and reach their asymptote, leaving fewer trials for the calculation of inspection times.

#### Magnetic resonance imaging

All children were imaged on a 3T Siemens Trio scanner, equipped with a 12-channel head RF array during natural, *non-sedated* sleep, or, if tolerated by the older children, while watching a favorite movie. Age-optimized mcDESPOT protocols comprise series of spoiled gradient recalled echo (SPGR) images and fully-balanced steady-state free precession (bSSFP) images acquired over a range of flip angles [33]. Inversion-prepared (IR-)SPGR data were also acquired to correct for transmit magnetic field (i.e., B_1_ field) inhomogeneities [38]; and the bSSFP data were acquired with two different phase-cycling patterns to allow correction for main magnetic field (i.e., B_0_ field) inhomogeneities [38]. All parameters were exactly the same as in [38]. A constant voxel dimension of 1.8 x 1.8 x 1.8mm^3^ was used for all children, with the field of view and imaging matrix adjusted depending on age and head size. Total image acquisition was less than 30 minutes for each child. To minimize acoustic noise, the maximum imaging gradient slew rates and peak values were reduced, and passive measures, including a sound-insulating bore liner, MiniMuff ear pads, and sound-attenuating ear protectors were used [39]. All images were visually inspected for motion related artifacts (ghosting, blurring, etc..) and data from all children were found to be usable.

Following acquisition, each child’s raw SPGR, IR-SPGR and bSSFP images were linearly co-registered to the high-flip angle SPGR image in order to account for subtle intra-scan motion [40], and non-brain signal was removed [41]. B_0_ and B_1_ field calibration maps were then calculated; followed by VF_M_ map calculation through the iterative fitting of a three-pool tissue model using a constrained fitting approach that provides stable estimates [42].

Following VF_M_ map calculation, each child’s map was non-linearly co-registered to a common study-specific template space for analysis. Described in more detail previously [33], the high flip angle T_1_-weighted SPGR image acquired as part of mcDESPOT is used to align the subject to this study-specific space. The calculated transformation matrix is then applied to the corresponding VF_M_ map. Registrations were assessed to ensure all VF_M_ maps were aligned to the study-specific template. Once all VF_M_ maps were transformed to the study-specific space, they were smoothed with a 4mm full-width-at-half-maximum 3D Gaussian kernel applied within a white and gray matter mask in order to account for subtle individual variations not accounted for by the registration procedure.

**Fig 2:**
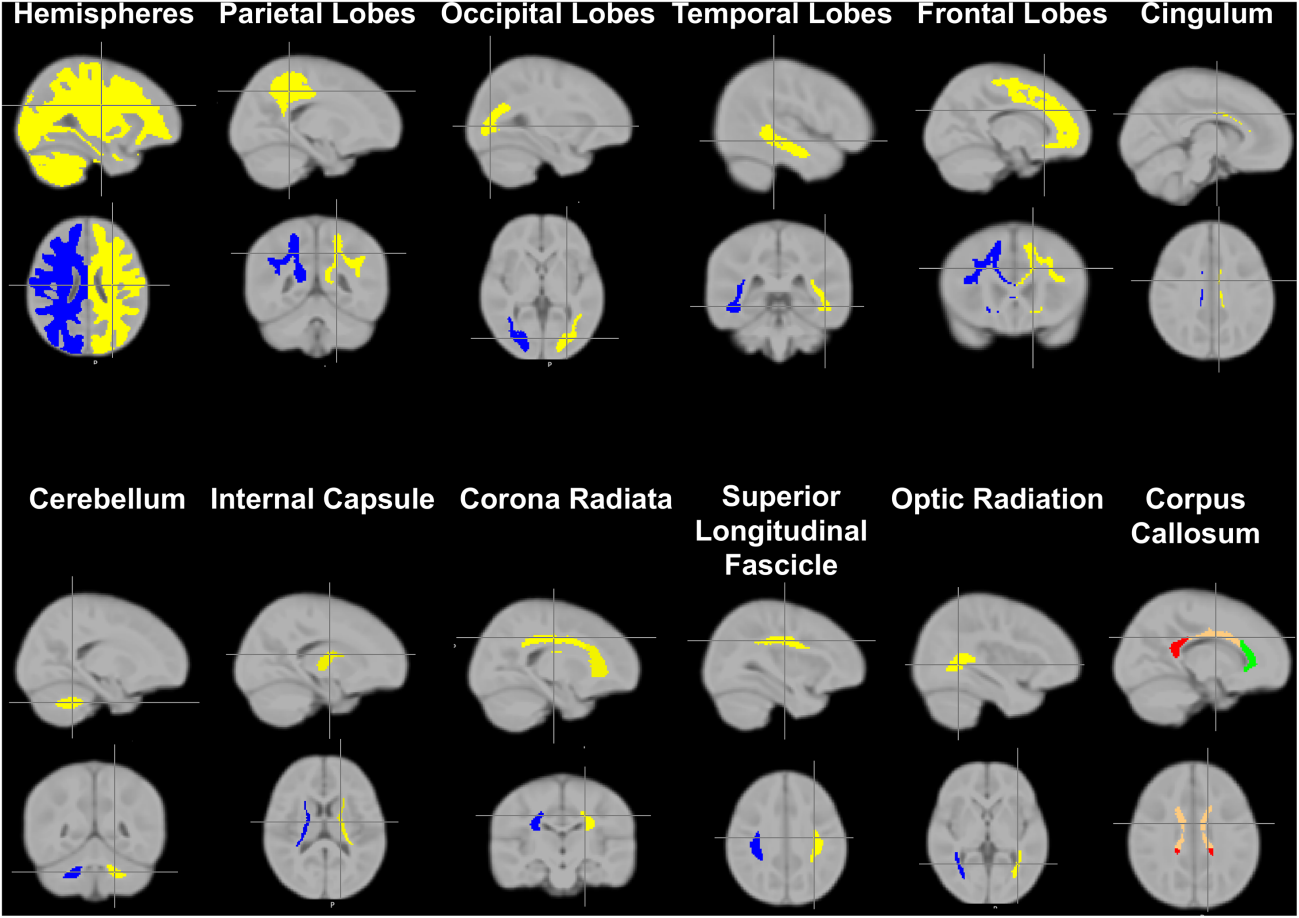
Predefined brain regions, for which VF_M_ was measured using the mcDESPOT [multicomponent Driven Equilibrium Single Pulse Observation of T_1_ and T_2_], white matter imaging technique.

Finally, mean VF_M_ values were obtained for the genu, splenium, and body of the corpus callosum; right and left hemisphere cingulum, corona radiata, internal capsule, and right and left hemisphere cerebellar, frontal, occipital, parietal, and temporal white matter regions (Fig 2). Atlases from the FMRIB Software Library (FSL; http://fsl.fmrib.ox.ac.uk) were used to obtain anatomical white matter masks (cerebellar, frontal, occipital, parietal, and temporal white matter) from the 2 mm resolution MNI template [43], while white matter tract masks (genu, splenium, and body of corpus callosum, cingulum, corona radiata, and internal capsule) were acquired from the John Hopkins University DT-MRI white matter atlas available within FSL [44]. Regional masks were brought into the study-specific template space by calculating the transformation from the MNI space to the study-specific space [33]. It has previously been shown that the normalization procedure described does not result in inhomogeneous alterations of mean VF_M_ values in template space compared with those in native-space [45 **]** and thus mean VF_M_ values were extracted from each child’s normalized VF_M_ map. Masks co-registered to the study-specific template were then superimposed onto each individual VF_M_ map and the mean and standard deviation VF_M_ for each region was calculated. Only voxels with VF_M_ greater than 0.001 were used in the calculation of regional means and standard deviations [33].

#### Statistical analysis

The relation between inspection times and VF_M_ was examined in two steps, following [16]. First, Pearson correlations between inspection times and VF_M_ were run separately to identify the brain regions that showed a significant relation to inspection times. As myelin volume increases across development [32,33], a second series of partial correlations between inspection times and VF_M_ was run controlling for age. Second, a linear hierarchical regression was performed to examine whether VF_M_ in any specific brain regions predicted inspection times over and beyond the influences of age and of VF_M_ in the other brain regions. Age was entered first in the hierarchical regression, whereas all the VF_M_ measures retained based on the correlation analyses were entered second.

## Results

**Fig 3:**
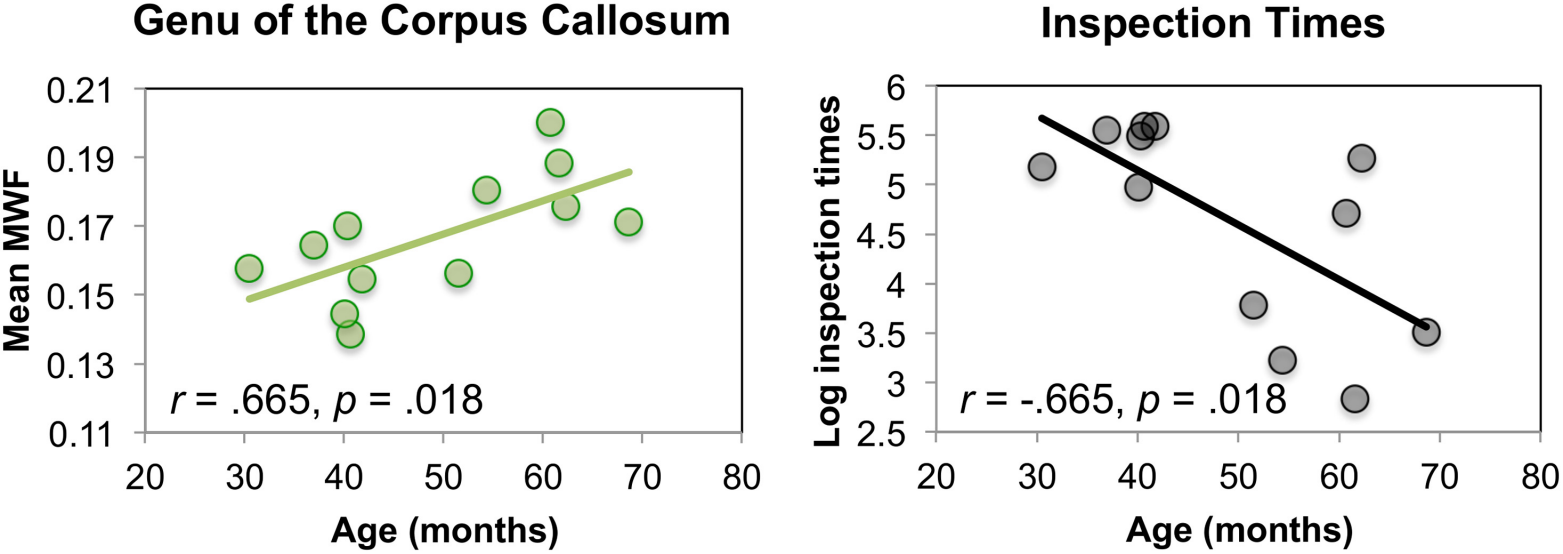
Significant Pearson correlations of age [in months] with inspection times [ln ms] and myelin volume fraction (VF_M_) in the genu of the corpus callosum.

Descriptive statistics for inspection times and mean VF_M_, and Pearson correlations are provided in Table 1 and Fig 3. Inspection times were negatively correlated with age, with older children needing shorter presentation times to process the target successfully, *r* = -0.665, *p* = 0.018. VF_M_ in the genu of the corpus callosum was also greater with age, *r* = 0.665, *p* = 0.018. Similar trends were observed for the body of the corpus callosum, the left and right corona radiata, the left internal capsule, the right cingulum, and the left frontal lobe, although they did not reach significance, all *p*s < 0.10.

**Table 1:** Descriptive statistics for age, inspection times and myelin volume fraction (VF_M_), raw Pearson correlations with age or inspection times, and partial correlations with inspection times (controlling for age).

	*M*	*SD*	Correlation with age		Correlation with PS		Partial correlation with inspection time	
			*r*	*p*	*r*	*p*	*r*	*p*
Age (in months)	49.1	12.3			**-.665**	**.018**		
Inspection time (ln ms)	4.64	1.02	**-.665**	**.018**				
VF_M_								
L. Frontal Lobe	.130	.013	.511	.089	-.464	.129	-.193	.570
R. Frontal Lobe	.130	.013	.360	.250	-.383	.219	-.205	.545
L. Parietal Lobe	.137	.011	.396	.203	-.412	.184	-.216	.523
R. Parietal Lobe	.137	.011	.385	.216	-.562	.057	-.444	.171
L. Occipital Lobe	.132	.015	.128	.692	**-.593**	**.042**	**-.685**	**.020**
R. Occipital Lobe	.141	.012	.036	.912	-.480	.114	**-.612**	**.046**
L. Temporal Lobe	.134	.014	.222	.489	-.447	.146	-.411	.209
R. Temporal Lobe	.139	.015	.103	.750	-.477	.117	-.550	.079
L. Cerebellum	.152	.010	-.226	.480	.117	.718	-.046	.893
R. Cerebellum	.163	.020	-.357	.254	-.195	.543	**-.621**	**.042**
L. Cingulum	.144	.020	.387	.214	-.263	.408	-.009	.980
R. Cingulum	.146	.020	.513	.088	-.544	.068	-.316	.345
L. Corona Radiata	.165	.014	.555	.061	-.553	.062	-.296	.377
R. Corona Radiata	.161	.015	.557	.060	-.515	.087	-.233	.490
L. Internal Capsule	.154	.014	.504	.095	**-.672**	**.017**	-.523	.099
R. Internal Capsule	.153	.016	.433	.159	**-.677**	**.016**	-.577	.063
L. Optic Radiation	.156	.014	-.077	.811	-.227	.477	-.375	.256
R. Optic Radiation	.156	.014	-.033	.918	-.282	.374	-.408	.213
L. SLF	.166	.016	.343	.275	-.424	.170	-.279	.406
R. SLF	.173	.015	.328	.298	**-.593**	**.042**	-.531	.092
Body of the CC	.147	.018	.538	.071	-.453	.140	-.150	.659
Splenium of the CC	.161	.014	-.120	.710	-.373	.232	**-.611**	**.046**
Genu of the CC	.166	.017	**.665**	**.018**	-.482	.112	-.071	.835

VF_M_ = myelin volume fraction. M = Mean. SD = Standard Deviation. L. = Left. R. = Right. SLF = Superior longitudinal fasciculus. CC = Corpus callosum. Significant correlations appear in bold.

**Fig 4:**
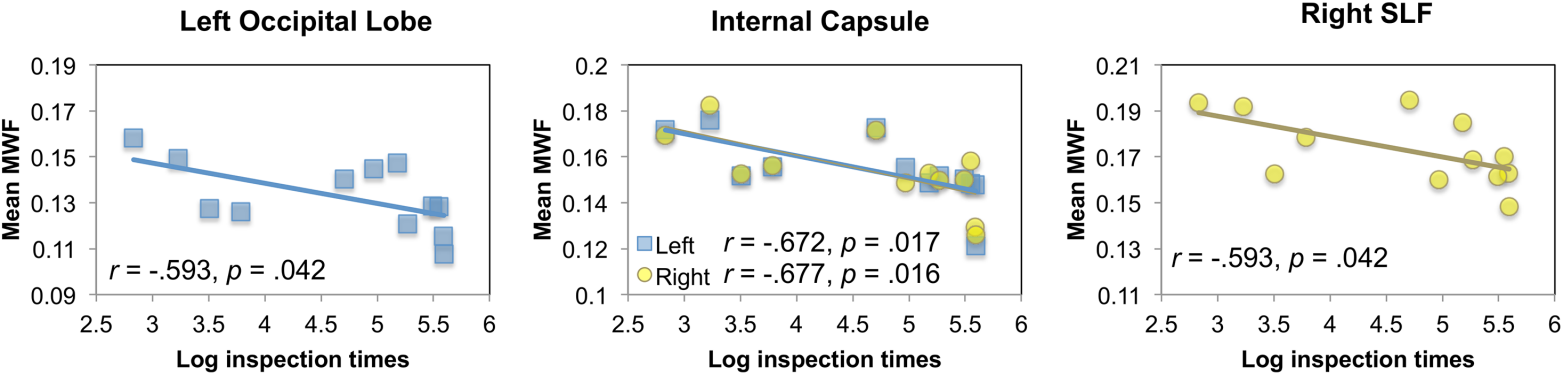
Significant Pearson correlations between inspection times [ln ms] and myelin volume fraction (VF_M_) in the left occipital lobe, left and right internal capsules, and right superior longitudinal fasciculus (SLF), before controlling for age.

Inspection times significantly correlated with VF_M_ in the left occipital lobe, left and right internal capsules, and the right superior longitudinal fasciculus, all *p*s < 0.05 (Table 1 and Fig 4). In addition, similar trends were observed for VF_M_ in the right parietal lobe, right cingulum, and left and right corona radiata, all *p*s < 0.10. Children showing greater VF_M_ in these regions needed less time to successfully identify the target. After controlling for age, the correlations were significant with myelin in the right and left occipital lobes, right cerebellum, and the splenium of the corpus callosum, all *p*s < 0.05, with additional trends for the right temporal lobes, left and right internal capsules, and right superior longitudinal fasciculus, *p*s < 0.10. However, with this small sample size, none of these correlations survived Benjamini and Hochberg [46] False Discovery Rate (FDR) corrections.

All brain regions showing significant partial correlations with inspection times were entered together in the hierarchical regression, after entering age (stepwise method). Correlations between the VF_M_ values entered in the regression ranged from *r* = .495 to *r* = .671 (Table 2) and variance inflation factors (VIF) were all below 2.57, which is lower than the conventional thresholds (.70 and 10, respectively) above which multi-collinearity may be a concern (e.g., [47]). Age was a significant predictor of processing speed performance, β = -0.665, *t*(11) = -2.817, *p* = 0.018, Adjusted *R* ^2^ = 0.387, *F*(1, 10) = 7.936, *p* = 0.018. Most importantly, when VF_M_ measures were added, VF_M_ in the left occipital lobe was retained in the model, β = -0.516, *t*(11) = -2.823, *p* = 0.020, resulting in a significantly better model, Adjusted *R* ^2^ = 0.639, *F*(2, 9) = 10.719, *p* = 0.004, *R* change = 0.262, *F*(1, 9) = 7.971, *p* = 0.020. The same results held when the analysis was run with VF_M_ in all the brain regions significantly correlated with inspection times before controlling for age. Therefore, VF_M_ in the left occipital lobe significantly predicted inspection times over and beyond the effect of age and of myelin in the other brain regions.

**Table 2:** Correlations among all myelin volume fraction (VF_M_) values.

	2	3	4	5	6	7	8	9	10	11	12	13	14	15	16	17	18	19	20	21	22	23
1. L. Frontal Lobe	**.839**	**.873**	**.752**	.428	.563	.501	.551	.138	-.051	**.728**	.559	**.976**	**.853**	**.769**	**.656**	.388	.295	**.628**	**.649**	**.676**	.286	**.829**
2. R. Frontal Lobe		**.696**	**.810**	.344	**.766**	.303	**.649**	.239	.180	.755	.582	**.859**	**.804**	**.673**	**.731**	.246	.369	.329	**.678**	.497	.280	**.803**
3. L. Parietal Lobe			**.656**	**.655**	.547	.664	**.622**	.359	.070	**.705**	.359	**.881**	**.782**	**.847**	**.671**	**.717**	.353	**.672**	**.708**	**.678**	.465	**.821**
4. R. Parietal Lobe				.475	**.792**	.494	**.709**	.159	.329	.533	**.616**	**.842**	**.766**	**.739**	**.695**	.270	**.733**	.557	**.781**	**.585**	.516	**.650**
5. L. Occipital Lobe					.560	**.831**	**.747**	.559	.514	.259	.192	.496	.594	**.780**	**.780**	**.870**	.561	**.670**	**.764**	.467	**.671**	.498
6. R. Occipital Lobe						.489	**.797**	.369	.538	.411	.399	.659	.566	**.627**	**.653**	.351	**.596**	.272	**.711**	.197	.420	.463
7. L. Temporal Lobe							**.614**	.534	.242	.105	.261	**.582**	**.635**	**.765**	**.644**	**.711**	**.634**	**.829**	**.699**	.480	.362	.482
8. R. Temporal Lobe								**.623**	**.770**	**.598**	.079	**.613**	**.756**	**.748**	**.755**	**.576**	**.643**	.469	**.910**	.473	.493	**.639**
9. L. Cerebellum									.504	.243	-.35	.153	.412	.271	.336	.575	.332	.196	.476	.005	.131	.372
10. R. Cerebellum										.211	-.291	.015	.232	.289	.38	.372	.568	.091	.572	.100	.495	.076
11. L. Cingulum											.147	**.674**	**.693**	.552	.519	.300	.051	.202	**.627**	**.614**	.269	**.827**
12. R. Cingulum												**.619**	.432	.483	.567	-.011	.313	.416	.343	.461	.296	.396
13. L. Corona Radiata													**.879**	**.846**	**.726**	.405	.429	**.665**	**.715**	**.680**	.325	**.839**
14. R. Corona Radiata														**.864**	**.843**	.464	.509	**.703**	**.830**	**.744**	.281	**.924**
15. L. Internal Capsule															**.895**	.670	**.590**	**.797**	**.847**	**.777**	.505	**.794**
16. R. Internal Capsule																**.583**	.554	**.640**	**.856**	**.688**	.526	**.774**
17. L. Optic Radiation																	.414	**.631**	.56	.443	**.657**	.428
18. R. Optic Radiation																		**.653**	**.663**	.439	.578	.250
19. L. SLF																			**.632**	**.772**	.471	.514
20. R. SLF																				**.722**	.566	**.748**
21. Body of the CC																					.501	**.714**
22. Splenium of the CC																						.210
23. Genu of the CC																						

VF_M_ = myelin volume fraction. L. = Left. R. = Right. SLF = Superior longitudinal fasciculus. CC = Corpus callosum. Significant correlations appear in bold (all *r*s ≥ .576 are significant, *p*s < .050).

## Discussion

The present study explored the relationship between processing speed, as measured by inspection times, and white matter myelin between 2 and 5 years of age. Both inspection times and VF_M_ correlated with age, with older children showing faster inspection times and greater VF_M_, hence providing further evidence for processing speed and myelination development in early childhood. Critically, VF_M_ in the left occipital region significantly predicted inspection times, beyond the effect of age. This finding is consistent with the hypothesis that white matter myelin contributes to processing speed early in development and this contribution is not a mere byproduct of age. Although the present study is a preliminary investigation, and more work is needed to test whether our findings hold when correcting for multiple comparisons in a larger sample, our findings are notable given the early age range, and given that we employed a new imaging method that more directly estimates myelin and inspection times, a measure of processing speed that minimizes other cognitive demands such motor response execution and goal maintenance.

Inspection times were associated with VF_M_ in the right and left occipital lobes, after controlling for age. Indeed, VF_M_ in the left occipital lobe predicted inspection times over and beyond age and VF_M_ in the other brain regions. It was the only significant predictor retained in the hierarchical regression after entering age, suggesting that it was key to inspection times. As occipital brain regions support visual information processing, this result brings further support to the claim that inspection times primarily reflect the perceptual processing speed of visuospatial information, rather than the speed of motor responding. Less expected was the stronger link between inspection times and VF_M_ in the left occipital lobe, relative to the right occipital lobe, although both correlated with processing speed beyond the effect of age. Fractional anisotropy in the left occipital lobe has similarly been found to relate to rapid picture naming and lexical access in school-age children and adolescents, perhaps reflecting faster access of language networks to visual information [15]. Although language does not seem particularly critical to visual inspection times, especially given that no overt verbal response was required in this task, verbal strategies may have helped children focus on critical target features (e.g., by labeling a visual feature such as the animal ears) that may have speeded up target processing. However, the specificity of the relationship between VF_M_ in the left occipital lobe and inspection times should be interpreted with caution, as VF_M_ in the right occipital lobe may have also turned out to be a significant predictor with a larger sample.

The association between VF_M_ in the occipital lobes and inspection times in preschoolers is consistent with findings later in development. Yet, unlike the present findings, those studies generally reported that myelin in frontal and parietal brain regions also significantly related to processing speed [48–52]. This apparent discrepancy may stem from differences across studies in the tasks used to assess processing speed. In most previous studies, processing speed tasks included high motor demands, given speed was mostly indexed via reaction times, as well as high demands on cognitive control [9]. Such motor and executive demands, which do not influence inspection times (or only minimally), may drive the relationship between processing speed and myelin in anterior regions. Alternatively, recent findings suggest that executive control abilities are not distinguishable from processing speed early in childhood and start separating by the end of the preschool period [7]. Because prefrontally mediated executive processes are still emerging, young children may mostly draw upon lower-order, bottom-up and task-specific processes to perform cognitive activities, while older children’s performance may rely more on top-down, executive processes. Such a progressive shift to prefrontally mediated executive processes may account for the increasing link between processing speed and myelin in anterior regions with advancing age. This would be consistent with the posterior-to-anterior pattern of brain development, with sensory cortex developing earlier than associative cortex (e.g., [53,54]). If so, inspection times may relate to VF_M_ in the prefrontal lobes in older children, whereas no such relationship should be observed if motor and executive demands, which are minimal for inspection times, drive the relationship with myelin in anterior regions.

Our findings should be considered in light of their limitations, in particular, the relatively small sample size that may hamper their generalizability. This study examined novel questions with challenging methodologies to address the role of myelin content for cognitive development. We used the most advanced technological approach to obtain quantitative measures of brain myelin content. Imaging research in toddlers is challenging for subjects, families, and investigators considering MRI-related space and movement constraints. We thus believe that this small but unique database is a rich source for preliminary insights. Yet, with a larger sample and greater statistical power, myelin in other brain regions may have also significantly predicted children’s inspection times.

Furthermore, processing speed was measured through inspection times only. It is plausible, and even probable, that myelin in brain regions other than the occipital lobe would relate to processing speed in tasks that do not as heavily rely on visual perception but on other cognitive demands. Conversely, faster inspection times with age may not be related to myelin exclusively but may also to the refinement of the neural networks and the number of brain regions supporting performance. In addition, the cross-sectional nature of our design prevents drawing strong conclusion on how myelination relates, or even may drive, changes in inspection times with age. Longitudinal investigations are needed to clarify this question. Finally, besides myelin, other brain changes not studied here, such as synaptogenesis, may influence inspection times and, more generally, processing speed early in childhood, and should be investigated in future.

Despite these limitations, our findings represent an important first step in the exploration of the brain bases of processing speed in early childhood. Establishing this link early in development is an important initial step to examine the potential role of myelination in cognitive development, as predicted by developmental cascade theories. Building on these findings, a major next step will consist in examining whether myelination influences cognitive abilities that lie further down in the developmental cascade, such as executive function, working memory, and intelligence, and even more distally, academic skills, and whether this effect is mediated by processing speed.

## Supporting Information

S1 Dataset (XLS)
